# Evidence of a New MoYpd1p Phosphotransferase Isoform in the Multistep Phosphorelay System of *Magnaporthe oryzae*

**DOI:** 10.3390/jof7050389

**Published:** 2021-05-15

**Authors:** Sri Bühring, Alexander Yemelin, Thomas Michna, Stefan Tenzer, Stefan Jacob

**Affiliations:** 1 Institute for Biotechnology and Drug Research gGmbH, Hanns-Dieter-Hüsch-Weg 17, 55128 Mainz, Germany; buehring@ibwf.de (S.B.); yemelin@ibwf.de (A.Y.); 2 Institut für Immunologie, Universitätsmedizin der Johannes-Gutenberg Universität Mainz, Langenbeckstr. 1, 55131 Mainz, Germany; thmichna@uni-mainz.de (T.M.); tenzer@uni-mainz.de (S.T.)

**Keywords:** phosphorelay, high osmolarity glycerol (HOG) pathway, alternative splicing, signaling, histidine kinases, *Magnaporthe oryzae*, *YPD1*, phosphotransfer, signal transduction

## Abstract

Different external stimuli are perceived by multiple sensor histidine kinases and transmitted by phosphorylation via the phosphotransfer protein Ypd1p in the multistep phosphorelay system of the high osmolarity glycerol signaling pathway of filamentous fungi. How the signal propagation takes place is still not known in detail since multiple sensor histidine kinase genes in most filamentous fungi are coded in the genome, whereas only one gene for Ypd1p exists. That raises the hypothesis that various Ypd1p isoforms are produced from a single gene sequence, perhaps by alternative splicing, facilitating a higher variability in signal transduction. We found that the mRNA of *MoYPD1* in the rice blast fungus *Magnaporthe oryzae* is subjected to an increased structural variation and amplified putative isoforms on a cDNA level. We then generated mutant strains overexpressing these isoforms, purified the products, and present here one previously unknown MoYpd1p isoform on a proteome level. Alternative splicing was found to be a valid molecular mechanism to increase the signal diversity in eukaryotic multistep phosphorelay systems.

## 1. Introduction

Signal perception, transduction, and, consequently, the response to environmental changes is a prerequisite for fungal biology and controls essential pathways, such as stress response, differentiation processes, metabolism, and pathogenicity [1]. The high osmolarity glycerol (HOG) pathway consists of a multistep phosphorelay (MSP) system connected to a conserved mitogen-activated protein kinase (MAPK) cascade [2]. Thereby, reversible phosphorylation and dephosphorylation are the molecular mechanisms to transmit extracellular stimuli to transportable intracellular signals and, thus, enable cellular adjustment or adaptation [3]. Apart from different biological functions, for example, cell wall integrity, heat stress, and plant infection [4], the major role of the HOG pathway is to regulate osmoadaptation in fungi [5]. Increasing extracellular osmotic stress results in the reduction of the phosphorylation events in the MSP, thereby activating the MAPK cascade downstream. The MAPK Hog1p translocates from the cytoplasm into the nucleus and regulates gene expression in order to adapt to external changes. As a result, intracellular accumulation of osmolytes compensates for the extracellular osmotic pressure to maintain cellular homeostasis [5]. Osmotic stress in the MSP of the model yeast *Saccharomyces cerevisiae* is perceived by the sensor histidine kinase (HK) Sln1p and transmitted through the histidine-containing phosphotransfer (HPt) protein Ypd1p to the response regulator Ssk1p [6]. Thus, Ypd1p has the important role of shuttling the signals from the sensors to the regulators and effectors. Sensor HKs are widespread in the fungal kingdom and encoded by multiple genes. However, the specific roles of HKs in the regulation of physiological processes are not entirely elucidated [7]. In addition to *Alternaria brassicicola*, *Aspergillus nidulans*, *Botrytis cinerea*, *Fusarium oxysporum,* and *Neurospora crassa*, the rice blast fungus *Magnaporthe oryzae* (anamorph *Pyricularia oryzae*) is an important model organism for researching the HOG pathway [8,9,10,11].

**Figure 1:**
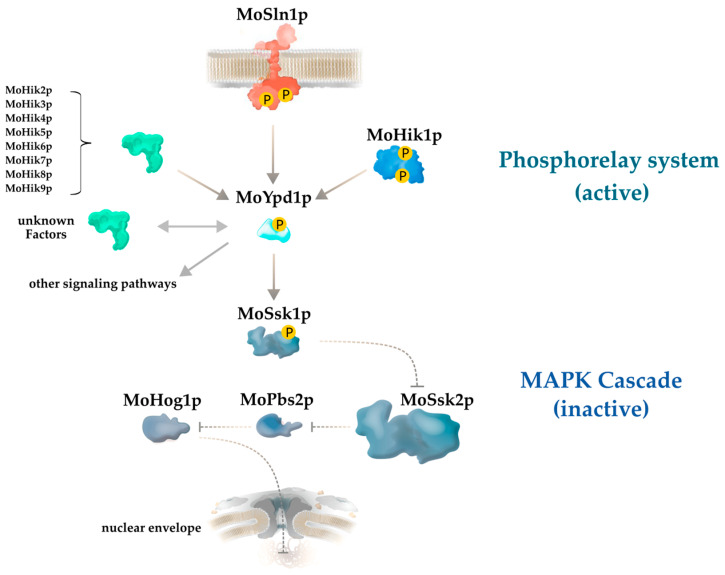
Simplified model of the central role of the phosphotransfer protein MoYpd1p in the high osmolarity glycerol (HOG) pathway of *Magnaporthe oryzae*. MoYpd1p transmits phosphorylation signals from at least two histidine kinases (HKs), MoSln1p and MoHik1p, to the response regulator protein MoSsk1p. MoSsk1p regulates the MAPK cascade MoSsk2p-MoPbs2p-MoHog1p. Putative protein interactions of MoYpd1p with the HKs MoHik2p–MoHik9p, unknown proteins/factors, or other signaling pathways are indicated with arrows.

The genome of *M. oryzae* contains ten genes for different sensor HKs (*MoSLN1*, *MoHIK1-9*), but only a single gene for a phosphotransfer protein is annotated, called *MoYPD1* (EnsemblFungi, [12]). It appears remarkable that, despite the ten HK-coding genes and the necessity of the coordinated transfer of phosphates within MSP systems, only one *YPD1*-like gene for the phosphotransfer protein is annotated in the genome. Protein–protein interaction of MoYpd1p with two of the HKs (MoSln1p, MoHik1p) is experimentally proven [8], whereas further interactions with the remaining eight HKs (MoHik2p-9p) and, consequently, networks to other signaling pathways are strongly suspected [8,12] (Figure 1).

This raises the fundamental question of how such variability in signal transduction via MoYpd1p is possible. A thoroughly reasonable explanation may be alternative splicing (AS), which has not been studied to a great extent for molecular mechanisms of signaling in fungal species. Alternative splicing is a molecular mechanism during the transcription process which has a vital role in eukaryotic gene regulation and increases the complexity of protein diversity [13]. Different isoforms with distinct biological functions are produced from precursor mRNA (pre-mRNA) by removing non-coding regions (introns) and joining coding regions (exons) to form mature mRNA [13]. Such pre-mRNA modifications enable one single gene to code for multiple different proteins. Apart from primary protein structure, intracellular localization and enzymatic function are also affected [14]. An impressive example of extraordinary protein diversity as a result of AS is the gene *Dscam* in *Drosophila melanogaster*, which contains 95 exons and, therefore, theoretically codes for more than 38,000 isoforms [15].

The broad spectrum of spliced mRNA and, consequently, the vast diversity of corresponding proteins illustrates the strong impact of AS on basic regulatory processes and signaling mechanisms within eukaryotic organisms. Whereas almost 95% of human multi-exon genes are alternatively spliced [16,17], the genomes of fungal species have remarkably fewer introns and fewer AS events, but this in no way reduces its importance [13]. However, the biological impact of AS on the molecular mechanisms of cellular signaling in fungi remains mostly unexplored. Recent reports indicate a much higher quantity of alternatively spliced mRNA products in fungi than previously assumed. In comparison to the low ratio of splicing events in *S. cerevisiae* (1.8%), an unexpected higher number of splicing events were assumed in filamentous fungi, such as *Shiraia bambusicola* (38.88%) and *Trichoderma longibrachitum* (76.2%) [18,19,20]. In this respect, according to the current genome annotation of *M. oryzae*, two transcripts for *MoYPD1* (MGG_07173T0, MGG_07173T1) are predicted (EnsemblFungi), based on in silico data. Interestingly, a more detailed analysis of our RNAseq datasets revealed significantly more structurally different *MoYPD1* transcripts [21]. Consequently, we analyzed these *MoYPD1*-associated sequences comprehensively on a DNA, RNA, and protein level. Furthermore, we generated a mutant strain overexpressing putative MoYpd1p isoforms and isolated and purified the products followed by LC-MS/MS analysis. In the end, we are able to present here experimental evidence of at least one new MoYpd1p isoform on a protein level.

## 2. Materials and Methods

### 2.1. Strains, Culture/Growth Conditions

The fungal strain used in this study was *M. oryzae* 70–15, respectively, *∆Moypd1* [8]. The strain was grown at 26 °C on complete medium (CM) at pH 6.5, agar (2%) containing per liter: glucose (10 g), yeast extract (1 g), peptone (2 g), casamino acids (1 g), nitrate salt solution (50 mL; containing per liter: NaNO_3_ (120 g), KCl (10.4 g), KH_2_PO_4_ (30.4 g), MgSO_4_ (10.4 g) × 7 H_2_O) and a trace element solution (1 mL; containing per liter: ZnSO_4_ (22 g) × 7 H_2_O, H_3_BO_3_ (11 g), MnCl_2_ (5 g) × 4 H_2_O, FeSO_4_ (5 g) × 7 H_2_O, CoCl_2_ (1.7 g) × 6 H_2_O, CuSO_4_ (1.6 g) × 5 H_2_O, Na_2_MoO_4_ (1.5 g) × 2 H_2_O and Na_2_EDTA (50 g), at pH 6.5 adjusted by 1 M KOH). Minimal medium (pH 6.5) contains per liter: glucose (10 g), a 0.01% biotin solution (0.25 mL), nitrate salt solution (50 mL), a trace element solution (1 mL) and a 1% thiamine dichloride solution (1 mL).

All chemicals used were pro analysis quality unless otherwise stated.

### 2.2. RNA Extraction and Quality Control, cDNA Synthesis

Mycelium of three-day-old liquid cultures was harvested, lyophilized, and ground into a fine powder under liquid nitrogen. Total RNA was isolated using the RNeasy^®^ plant mini kit (Qiagen, Hilden, Germany), complying with the manufacturer’s instructions. The LunaScript^®^ RT SuperMix Kit (New England Biolabs Inc., Ipswich, MA, USA) was used for cDNA synthesis, following the manufacturer’s instructions. The RNA integrity numbers and DNA contaminations were assessed by an Agilent 2100 bioanalyzer (Agilent Technologies, Santa Clara, CA, USA) using the RNA 6000 Nano Kit (Agilent Technologies). High-quality RNA with RNA integrity numbers values above 8 were used for the transcriptome analysis.

### 2.3. PCR of MoYPD1 Isoforms on cDNA Level

A polymerase chain reaction (PCR) was performed in a total volume of 50 µL containing 5 µL of each primer (100 pmol/µL), 1 µL dNTPs (10 mM)) 2 µL cDNA, 10 µL 5× Phusion HF-Buffer, 0.5 µL Phusion Hot Start II DNA Polymerase (ThermoFisher Scientific, Waltham, MA, USA) and 26.5 µL RNase free water to fill the reaction. The cDNA amplification was performed in a C1000 Touch Thermal Cycler (BioRad Laboratories, Hercules, CA, USA), applying the following reaction conditions: initial denaturation for 30 s at 98 °C, 35 cycles consisting of denaturation at 95 °C for 10 s; annealing at 61 °C for 15 s, extension at 72 °C for 20 s, and a final extension step at 72 °C for 5 min. The PCR product was separated by electrophoresis in a 1% agarose gel (Biozym Scientific GmbH, Oldendorf, Germany), stained with ethidium bromide, and visualized using a QUANTUM-ST5-1100/26MX system (PEQLAB Biotechnologie GmbH, Erlangen, Germany).

### 2.4. DNA Manipulations, Construction of the Expression Vector, and Fungal Transformation

The DNA of *M. oryzae* was isolated from mycelium of three-day-old liquid cultures (grown in CM at 26 °C and 120 rpm) using the DNeasy^®^ Plant Mini Kit (Quiagen GmbH, Hilden), following the manufacturer’s instructions for the purification of DNA from plants and filamentous fungi. The DNA manipulation followed standard procedures [22]. NEB^®^ 10-β competent *Escherichia coli* strains (high efficiency) were used for the routine bacterial transformations and construction of plasmids.

Fungal transformation of *M. oryzae* was conducted using *Agrobacterium tumefaciens*-mediated transformation. The detailed procedures followed those described previously [21].

The plasmid used for fungal transformation is based on a backbone of a binary *Agrobacterium*-compatible vector *pSJ + GFP(BAR)* to generate the overexpression mutant *ΔMoypd1/EF1::YPD1(TS)* [23]. The mutants were generated by using a glufosinate–ammonium resistance (modified bialaphos resistance gene: BAR). The selection of transformants resistant to glufosinate–ammonium was performed using 100 µg mL^−1^ of the antibiotic in a minimal medium.

The plasmid used for fungal transformation of *ΔMoypd1/EF1::YPD1(TS)* was generated by the Gibson Assembly^®^ cloning method [24].

*pSJ + GFP(BAR/nTwinStrep)* was generated by amplifying the fragment EF1prom from *pSJ + GFP(BAR)* with the primers SJ-632/SJ-633 (Table 1) and the fragment GFP with the primers SJ-634/SJ-635 in order to create a flexible cloning plasmid with which the Twin-Strep-tag^®^ can be fused to the N-terminal of the gene of interest. The coding sequence for the Twin-Strep-tag^®^ (inclusive of an N-terminal ATG-startcodon and a C-terminal *Bgl*II-restriction side (A/GATCT) as a spacer) was included in the primer SJ-634. The *Sca*I/*Bsr*GI-restricted vector *pSJ + GFP(BAR)* was used as a backbone vector.

The 8674 bp expression-plasmid *pSJ + EF1::YPD1(BAR)* was generated by using the *Bgl*II/*Bsr*GI-restricted *pSJ + GFP(BAR/nTwinStrep)* as a backbone. The genomic sequence of *MoYPD1* (Locus MGG_07173) was amplified from genomic DNA with the primers SJ-651/SJ-652.

The plasmid *pSJ + EF1::YPD1(BAR)* was finally used to transform *∆Moypd1* via *Agrobacterium tumefaciens*-mediated transformation, resulting in the mutant strains *ΔMoypd1/EF1::YPD1(TS)*.

### 2.5. Protein Expression and Purification

The strain *ΔMoypd1/EF1::YPD1(TS)* was cultivated in 200 mL liquid CM in 500 mL glass flasks with one baffle for four days at 26 °C and 120 rpm. Mycelium was harvested by vacuum-filtrating the culture through filter paper (MN 615, MACHEREY-NAGEL), immediately frozen under liquid nitrogen, and freeze-dried. About 3 g of lyophilized mycelium of the expression strain *ΔMoypd1/EF1::YPD1(TS)* was used for the purification process. The samples were powdered with the TissueLyserII (Qiagen) using the Grinding Jar Set (S. Steel, 2 × 10 mL, with grinding balls, Qiagen), according to the manufacturer’s instructions. The adapter sets were precooled at −80 °C for at least 2 h. The powdered mycelium was dissolved in binding buffer (150 mM NaCl, 100 mM Tris-HCl pH 8, 1 mM EDTA, protease inhibitor cocktail (Sigma), 0.1%Tween 20), homogenized in an ultrasonic unit for 30 s, and then centrifuged for 45 min at 4 °C and 18,000 rpm. The supernatant was filtered (nitrocellulose membrane filter, 0.45 µm, Sartorius Stedim Biotech GmbH, Göttingen, Germany), degassed, and applied to 1 mL Streptactin XT gravity-flow columns for affinity purification. Loading, washing steps, and elution were carried out according to the manufacturer’s instructions. The BXT elution buffer (from IBA, with 50 mM biotin) was used for elution. Six fractions of 1 mL each were generated and selected fractions combined for the protein analysis.

### 2.6. In-Gel Tryptic Digest

Sixteen bands from the SDS-PAGE were excised and cut into 1 mm × 1 mm pieces and washed twice with 200 µL of 50 mM ammonium bicarbonate (AMBIC) in 50% acetonitrile (ACN) followed by a single wash with 200 µL pure ACN each by sonification for 5 min at room temperature, discarding the supernatant. The reduction of disulfide bonds was performed by the addition of 100 µL of 10 mM dithiothreitol in 50 mM AMBIC/50% ACN and incubation for 60 min at 56 °C. After discarding the supernatant, 100 µL of 55 mM iodoacetamide was added for alkylation of the reduced disulfide bonds by incubation for 45 min at room temperature, keeping the samples in the dark. The supernatant was discarded, and the gel pieces were washed twice with 200 µL of 50 mM AMBIC/50% ACN followed by a single wash with 200 µL pure ACN each by sonification for 5 min at room temperature, discarding the supernatant. The remaining ACN from the gel pieces was evaporated by warming the samples at 56 °C for 30 s. A proteolytic digest was performed by the addition of 2.5 µg Trypsin in 25 µL 50 mM AMBIC and incubation overnight at 37 °C. The resulting peptides were recovered by washing the incubated gel pieces twice with 50 µL 0.1% formic acid (FA) in 50% ACN and sonicating each for 15 min and combining the supernatants in a new sample tube. The peptides were frozen at −80 °C and lyophilized. The lyophilized peptides were reconstituted in 20 µL 0.1% FA for mass spectrometric analysis.

### 2.7. LC-MS/MS Peptide Identification

A volume of 2.6 µL of the peptide mixture was injected in an ultra-high performance liquid chromatography nanoAcquity system (Waters Corporation) equipped with a HSS-T3 C18 1.8 μm, 75 μm × 250 mm reverse-phase column (Waters Corporation) for each measurement. The separation of peptides was performed at 55 °C using a 90 min gradient from 5 to 40% mobile phase B consisting of 0.1% FA/3% DMSO in ACN, while mobile phase A consisted of 0.1% FA/3% DMSO in water. The flow rate was set to 300 nL/min, and all separations were followed by washing the column with 90% mobile phase B and sufficient re-equilibration. After elution from the chromatographic column, the peptides were ionized by electrospray ionization and subjected to mass spectrometric analysis using a Synapt G2-S HDMS QTOF mass spectrometer (Waters Corporation) which was operated in data-dependent acquisition mode with a typical resolving power of R = 20,000. The top ten most intense peptide precursor m/z from MS1 spectra were selected for further fragmentation in the quadrupole, followed by measurement of the resulting fragment m/z as MS2 spectra. (Glu1)-Fibrinopeptide B was injected as a lock mass into the flow path every 30 s via the reference sprayer of the NanoLockSpray source for post-acquisition mass correction. All samples were measured in triplicates.

### 2.8. Data Processing

The raw files were processed using PEAKS Xpro (Version 10.6, BSI, Canada). The m/z error tolerance was set to 15 ppm for parent ion and 0.03 Da for fragment ion matches. Trypsin was specified as a digesting enzyme in specific mode, allowing up to one missed cleavage. Carbamidomethylation (+57.02 Da) on cysteine was set as a fixed modification, and one oxidation (+15.99 Da) on methionine as a variable modification was allowed. A combined FASTA file was used for the database search consisting of, first, the uniport *M. oryzae* reference proteome (UP000009058) downloaded on 17 March, 2021, with 12,791 entries; second, 173 common contaminants and, third, two amino acid sequences from putative *YPD1* isoforms translated from possible open reading frames of cDNA sequenced previously. For confident peptide identification, all resulting peptides were filtered for 1% false discovery rate using a target decoy database, which corresponds to a −10 LgP score of 44.5 for each peptide spectrum match.

The mass spectrometry proteomics data have been deposited in the ProteomeXchange Consortium via the PRIDE (https://www.ebi.ac.uk/pride/archive, accessed on 5 May 2021) partner repository with the dataset identifier PXD024832.

## 3. Results

### A Novel MoYpd1p Isoform Enables Signal Diversity in the Multistep Phosphorelay System

**Figure 2:**
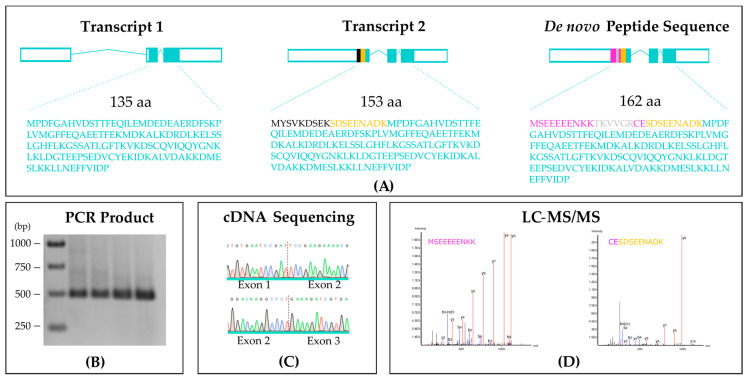
(**A**): Schematic representation of the mRNA transcripts of *MoYPD1,* including the translated open reading frames of the predicted proteins. Exons are depicted as solid boxes and introns as lines. Untranslated regions of the transcripts are represented as white boxes. Gray, yellow and pink highlights within exons and amino acid sequence show differences between the isoform sequences. Additional amino acids (methionine (M), serine (S), glutamic acid (E), cysteine (C), asparagine (N), and lysine (K) determined by de novo peptide sequencing are highlighted in pink. (**B**): Detection of the 489 bp cDNA amplicon with the primer pair SJ-955 and SJ-956 by electrophoresis. (**C**): Electropherograms of Sanger sequencing analysis of the 489 bp PCR product from (**B**), including exon junctions (dottet line). (**D**): De novo sequencing results of the tryptic peptide fragments MSEEEEENKK and CESDSEENADK by LC-MS/MS analysis.

We strove to identify new isoforms of *MoYPD1* in order to unravel the enigma of multiple signals and a limited number of signaling pathways [25]. Towards this purpose, we extracted RNA from *M. oryzae* grown on complete medium, which served as a template for reverse transcription into cDNA. We then amplified the cDNA using different primer pairs designed based on sequence predictions from the RNAseq data of [25] existing already. The forward primers should bind in the 5′ untranslated region and the reversed primer next to the 3′ stop-codon. We amplified several cDNA products, and we detected a 486 bp calculated PCR product by electrophoresis using the primer pair (SJ-955/SJ-956) (Figure 2B). After sanger sequencing of the purified cDNA-fragment (Figure 2C), an alignment with the previous sequence annotation of *MoYPD1* transcripts (EnsemblFungi MGG_07173T1 (408 bp) and MGG_07173T0 (462 bp)) revealed the presence of 27 additional nucleotides in the 5′ direction. These findings indicated a novel transcript-based annotated isoform of *MoYPD1* on the cDNA level. We predicted the open reading frames and translated them into amino acid sequences. Figure 2A shows the in silico protein translation of the two annotated (Transcript 1 and Transcript 2) and the previously unknown transcript of MoYPD1 (de novo peptide sequence). The amino acid sequences were predicted to be predominantly identical for the hypothetical proteins. The additional peptide MSEEEEENKKTKVVGRCESDSEENADK was predicted for the novel *MoYPD1* transcript in comparison to transcript *MGG_07173T1* (Transcript 1) and *MGG_07173T0* (Transcript 2), respectively.

We next generated overexpression mutants of MoYpd1p, called *ΔMoypd1/EF1::YPD1(TS)*, with an N-terminally attached Twin-Strep-tag^®^ by using the loss of function mutant *ΔMoypd1* as the parent strain to validate the new transcript-based in silico annotation on a protein level. The affinity tag was fused directly in front of one in silico predicted startcodon upstream in the 5′ untranslated region of the *MGG_07173T1* and *MGG_07173T0* annotations. That means, on the basis of this startcodon, isoforms would be transcribed and translated. After MoYpd1p-expression, affinity chromatography, and in-gel digestion, the peptide samples were analyzed by LC-MS/MS. As a result, two peptide fragments that were unique to the novel *MoYPD1* transcript were confidently identified with a −10 LgP identification score above the quality threshold of 44.5. The first peptide MSEEEEENKK (Figure 2A right side, pink letters) was identified by 41 peptide spectrum matches in gel bands 3–6 and 11–16 with charge states z = 2 and 3, a median −10 LgP score of 89.87, and a median parent mass error of 5.1 ppm. The second peptide with the sequence CESDSEENADK was identified by 32 peptide spectrum matches in gel bands 3–4 and 11, exclusively in charge state z = 2, a median −10 LgP score of 70.02, and a median parent mass error of 4.3 ppm. It is, in general, very hard to transform the null mutant *ΔMoypd1* because of its very strong phenotype (i.e., no spores, albino, fludioxonil resistant, and strong osmosensitivity [8]. We showed that reintegration of the original annotated gDNA sequence and the native promoter into the genome of *ΔMoypd1* resulted in a complemented strain *ΔMoypd1/YPD1* restored in some essential functions. The *ΔMoypd1/YPD1* phenotype had restored osmoregulation and was sensitive toward fludioxonil as compared to *ΔMoypd1* (osmosensitive and fludioxonil resistant), but it did not completely restore the WT phenotype in vegetative growth [8]. The *ΔMoypd1/EF1::YPD1(TS)* phenotype resulted in the same observations as for *ΔMoypd1/YPD1* in different stress-inducing ingredients, such as NaCl (salt stress), sorbitol, and fungicide treatment. Up to now, we have isolated the new isoform only from mycelium of *ΔMoypd1/EF1::YPD1(TS)*. Currently, we are on the way to look for different isoforms produced “signal-specific” or at different stages of the life cycle of the *M. oryzae* 70-15 strain.

To the best of our knowledge, this is the first report confirming this newly deduced *MoYPD1* transcript on a protein level (Figure 2D). Consequently, AS was found to be a valid molecular mechanism to increase signal diversity in eukaryotic MSP systems.

## 4. Discussion

Investigating signaling pathways to unravel the dogma of sensing multiple signals despite a limited number of pathway components might improve our current understanding of physiological processes in fungi. The phosphotransfer protein MoYpd1p is the key mediator within the phosphorelay system of the HOG pathway and enables signal transduction via phosphorylation and dephosphorylation in response to environmental changes. Despite MoYpd1p seeming to interact with multiple sensor HKs and at least one response regulator, only one coding gene *MoYPD1* exists in the genome of *M. oryzae* [8]. That raises the fundamental question of how the signaling variability of *MoYPD1* occurs: how can one phosphotransfer protein route and coordinate many different inputs to generate completely different output reactions? One idea of how signal transduction in phosphorelay systems with only one phosphotransfer protein could be achieved is AS [8]. To date, two transcripts of *MoYPD1* (*MGG_07173T0*, *MGG_07173T1*) have been annotated. Despite the fact that MoYpd1p is known to be a phosphotransfer protein, knowledge of the putative alternatively spliced isoforms and their interactions with other pathway components is scarce. We present here the first report of a previously unknown protein isoform of *MoYPD1,* by capturing evidence on both a cDNA and protein level. The cDNA of the newly identified transcript contains 27 nucleotides more than the annotated transcripts. We were able to confirm two unique peptides (MSEEEEENKK and CESDSEENADK, Figure 2A right side in pink) in accord with the newly discovered *MoYPD1* transcript by nanoUPLC-MS/MS. Of course, these additional N-terminal peptides could affect the 3D structure, folding, and, thereby, the phosphotransferase activity of MoYpd1p. Our results demonstrate clearly that more MoYpd1p isoforms exist than previously suggested, which may increase the signal diversity and provide the basis for further studies on signal transduction in fungal MSP systems. The discovery of the new isoform is consistent with recent studies which showed that AS events play a crucial role in the fungal kingdom [26,27]. The gene *MoGRP1*, for example, encoding a glycine-rich RNA-binding protein, was identified as a splicing factor, which is essential for the removal of introns in *M. oryzae*. The deletion of *MoGRP1* resulted in a reduced stress response, virulence, mycelial growth, and conidiation [27]. Unraveling the complex signaling mechanisms in fungi is complicated due to a lack of isoform annotation for most fungal genes [28]. Consequently, the annotations do not distinguish the functions of the different proteins derived from a single gene. A transcriptome analysis with *Verticillium dahlia* has shown that approximately 50% of multi-exonic genes are subjected to AS regulation, with 90% of splicing events due to intron retention. In addition, the study showed that a gene can be transcribed via four different 5′-splice sites and two different 3′-donor sites into five mature mRNAs [19]. Another example of AS as a key mechanism for protein diversity is known from *Sclerotinia sclerotiorum.* Isoforms merely expressed in planta by host-specific AS were identified in this phytopathogen. Thereby, colonization of diverse host families led to the accumulation of different transcripts and, consequently, altered protein domain structures, suggesting alternative functions of the isoforms during plant infection [29].

It is known from higher eukaryotes that signal transduction pathways, including proteins with reversible phosphorylation reactions, have a strong influence on isoform formation and are essential for developmental processes and environmental stress responses. Numerous genes, such as protein kinases, transcription factors, splicing regulators, and pathogen resistance genes, have been identified to regulate stress responses [30]. The expression of different transcript variants of the rice gene *OsBWMK1*, a MAPK family member, was detected in rice tissue. After various stress conditions, AS was shown to alter the domain architecture, affecting its subcellular localization but not its activity. Ypd1p homologs from *Candida albicans* and *S. cerevisiae* have previously been detectable in both cytoplasm and nucleus [31]. It is therefore conceivable that different isoforms of MoYpd1p exist in *M. oryzae*, altering the subcellular localization of the protein rather than the protein function. Up until now, little research has previously dealt with the AS of phosphotransfer-protein encoding genes and the physiological purpose of different phosphotransfer protein isoforms. In the current study, we presented the first step to study AS as a molecular mechanism of signal transduction diversity and demonstrated insights to answer some of the fundamental questions in biology: how diversity is achieved in signal transduction?

## Figures and Tables

**Table 1:** List of oligonucleotides used in this study.

Name	Sequence (5′→ 3′)
SJ-632 (EF1prom-for)	ttactgatcactgattaagtCTGAGAGCGAGAAAAAAAAACTCTTC
SJ-633 (EF1prom-rev)	tgaggatgactccacatGGTGGCGGTTTGGTGCTC
SJ-634 (GFP-for)	aaccgccaccatgtggagtcatcctcaattcgagaaaggtggaggttctggcggtggatcgggaggttcagcgtggagccacccgcagttcgaaaaagatctGTGAGCAAGGGCGAGGAG
SJ-635 (GFP-rev)	gccgggcggccgctttacttTTACTTGTACAGCTCGTCCATG
SJ-651(Ypd1-twinstrep-for)	gaggttcagcgtggagccacccgcagttcgaaaaaATGTCGGAGGAGGAGGAGGAGAACA
SJ-652(Ypd1-twinstrep-rev)	cgatctgcagccgggcggccgctttacttttacttCTAAGGATCGATCACGAAAAACTCATTG
SJ-955 (Ypd1-var3-rev)	CTAAGGATCGATCACGAAAAAC
SJ-956 (Ypd1-var4-for)	ATGTCGGAGGAGGAGGAG

## Data Availability

The mass spectrometry proteomics data have been deposited in the Proteo-meXchange Consortium via the PRIDE (https://www.ebi.ac.uk/pride/archive, accessed on 5 May 2021) partner re-pository with the dataset identifier PXD024832.
